# Microbiological Quality of Fresh Produce from Open Air Markets and Supermarkets in the Philippines

**DOI:** 10.1155/2014/219534

**Published:** 2014-05-13

**Authors:** Pierangeli G. Vital, Kris Genelyn B. Dimasuay, Kenneth W. Widmer, Windell L. Rivera

**Affiliations:** ^1^Institute of Biology, College of Science, University of the Philippines, Diliman, 1101 Quezon City, Philippines; ^2^Natural Sciences Research Institute, University of the Philippines, Diliman, 1101 Quezon City, Philippines; ^3^International Environmental Analysis and Education Center, Gwangju Institute of Science and Technology, 261 Cheomdan-Gwagiro, Buk-gu, Gwangju 500-712, Republic of Korea

## Abstract

This study is the first in the Philippines to conduct a comprehensive assessment of the prevalence of bacterial pathogens and somatic phages in retailed fresh produce used in salad preparation, namely, bell pepper, cabbage, carrot, lettuce, and tomato, using culture and molecular methods. Out of 300 samples from open air and supermarkets, 16.7% tested positive for thermotolerant *Escherichia coli*, 24.7% for *Salmonella* spp., and 47% for somatic phages. Results show that counts range from 0.30 to 4.03 log_10_ CFU/g for *E. coli*, 0.66 to ≥2.34 log_10_ MPN/g for *Salmonella* spp., and 1.30 to ≥3.00 log_10_ PFU/g for somatic phages. Statistical analyses show that there was no significant difference in the microbial counts between open air and supermarkets (*α* = 0.05). TaqMan and AccuPower Plus DualStar real-time polymerase chain reaction (RT-PCR) was used to confirm the presence of these organisms. The relatively high prevalence of microorganisms observed in produce surveyed signifies reduction in shelf-life and a potential hazard to food safety. This information may benefit farmers, consumers, merchants, and policy makers for foodborne disease detection and prevention.

## 1. Introduction

Food-borne pathogens take a serious toll on public health. It is estimated in the United States alone that approximately 14 million incidents of food related illness occur [1]. A recognized source for food-borne pathogens is fecal contamination of water used for irrigation, or for processing, of fresh produce [2]. While many agricultural products are cooked prior to eating, many Southeast Asian cultures also consume uncooked produce either directly or as fresh condiments to other dishes, such as soups.

Surveys of agricultural produce, meats, and shellfish have been conducted finding relatively high microbial loads in Southeast Asia [3, 4] indicating that contamination of water for agriculture and aquaculture, compounded by poor food handling during distribution, can have a negative impact on public health. Some have also investigated risk assessment models based on consumption of fresh produce [5]. While these survey studies have been conducted, actual sampling data is lacking for many developing countries making attempts to accurately develop risk assessment studies problematic. Some approaches to assess microbial risk associated with drinking water based on theoretical values for developing countries have been attempted [6]. Yet, the additional variables of the transfer of waterborne pathogens to produce through irrigation and washing of produce can make such theoretical values difficult to calculate. Other approaches involving direct sampling of produce for pathogens to evaluate microbial risk assessment have been employed [7]; however, sufficient comprehensive survey data of produce in Southeast Asia, particularly in the Philippines, is lacking.

Additionally, studies for the impact of enteric viruses on produce in the Philippines are also limited. As microbial contamination of agricultural products is of concern, additional survey data would be a key to assessing their impact on food safety [1]. A comprehensive survey for the presence and enumeration of bacterial pathogens, in addition to establishing the quantification of somatic bacteriophages as an indicator for viral pathogens, would be ideal for determining microbial contaminant loads on fresh produce used in salad preparation through culture and molecular methods. This study investigates the prevalence of thermotolerant* Escherichia coli*,* Salmonella *spp., and somatic bacteriophages in fresh produce consumed uncooked, namely, bell pepper, cabbage, carrot, lettuce, and tomato, found in both open air markets and supermarkets in the Philippines. This information would be a cornerstone to determining the risk associated with microbial contamination of freshly consumed foods for populations in the Philippines and safer vending practices. This study is the first in the country to address microbial contamination of fresh produce that may directly or indirectly benefit farmers, consumers, merchants, vendors, and policy makers towards food quality and safety.

## 2. Materials and Methods

### 2.1. Produce Sampling

A total of 300 fresh produce samples typical for raw consumption were surveyed. Sampling areas included five large local and open air markets where most residents and retailers purchase produce from National Capital Region, Laguna (province in South Luzon), and Pampanga (province in Central Luzon). Five common and known supermarkets were also surveyed to compare the microbial quality of produce from that of open air markets. Five types of vegetable produce that are consumed uncooked, namely, bell pepper (*Capsicum frutescens, *family Solanaceae), cabbage (*Brassica oleracea, *family Brassicaceae), carrot (*Daucus carota, *family Apiaceae), lettuce (*Lactuca sativa, *family Asteraceae), and tomato (*Lycopersicon esculentum, *family Solanaceae), were collected from a variety of sources to encompass the different produce handling and distribution practices. A total of 50 vegetables were collected per market, ten of each kind, and another 50 from supermarkets. All samples purchased were placed in individual polyethylene bags. Vegetable produce samples were transported to the Natural Sciences Research Institute, University of the Philippines, in an improvised ice box (kept under 10°C) and processed within 6–8 h after collection.

### 2.2. Produce Analysis: Culture Method

Using sterile scalpel blades, 5 g portion was excised from the samples, weighed in sterile plastic boats, and transferred to sterile whirl pack bags (Nasco Whirl-Pak, USA). Thirty millilitres of sterile 0.1% buffered peptone water (BPW) (MB Cell, Korea) was added; then bags were sealed and rotated on a shaking platform for 10 min. The produce wash was then processed for microbial analysis.

#### 2.2.1. Detection and Enumeration of* Escherichia coli*

After undergoing the washing step, the buffer was examined for the presence of thermotolerant* E. coli*, an opportunistic pathogen and classic indicator organism for fecal contamination, using a membrane filtration technique [8]. Briefly, tenfold serial dilutions were performed from the 10 mL wash solution in BPW. They were filtered through 0.45 *μ*m filters (PALL Corporation, USA), plated on membrane-fecal coliform (m-FC) agar plates (MB Cell, Korea), and incubated at 44.5 ± 0.2°C for 24 h where CFU/g of fresh produce was obtained. Blue to deep blue colonies were considered presumptive for* E. coli* and at least four isolated colonies were streak plated on MacConkey agar (MCA) plates (MB Cell, Korea) and incubated at 35 ± 0.5°C for 24 h. Streaked colonies were considered thermotolerant* E. coli* indicated by the presence of light pink or red colonies.

#### 2.2.2. Detection and Enumeration of* Salmonella* Strains

The wash buffer solution was also examined for the presence and enumeration of* Salmonella *spp. using MPN method in Rappaport-Vassiliadis Enrichment Broth (RV) (MB Cell, Korea). Briefly, 5 mL volume of the wash was mixed with 5 mL double strength BPW, and 3 further tenfold serial dilutions were performed in BPW as an enrichment step. All tubes were incubated at 35 ± 0.5°C for 24 h. Afterwards, 0.2 mL per dilution series was added to 1.8 mL RV in three respective wells (12 wells total) using a sterile multiwell plate and incubated at 42 ± 0.5°C for 24 h. Results were recorded as positive (+) or negative (−) and compared to an MPN table to obtain MPN/g of fresh produce. Positive wells were streak plated in triplicate onto xylose lysine deoxycholate (XLD) agar plates (Difco, Becton, Dickinson and Company, USA) and incubated at 35 ± 0.5°C for 18–24 h.* Salmonella* spp. isolates were indicated by red colonies with a dark center, confirming results from the RV broth MPN.

#### 2.2.3. Detection and Enumeration of Somatic Phages

The wash buffer was also tested for the presence of somatic bacteriophages. Somatic phages are classic indicator organisms of fecal contamination and have been demonstrated as efficient model organisms for viral pathogens [9–11]. A stock culture of* E. coli* CN-13, a nalidixic acid resistant strain, was maintained in tryptic soy agar (TSA) (MB Cell, Korea) with nalidixic acid and used to serve as somatic bacteriophage host for this assay. Portions of the sample wash were processed for somatic phage enumeration using a double agar layer method. Briefly, 0.7% tryptic soy broth (TSB) (MB Cell, Korea) soft agar tubes were prepared with nalidixic acid, and log host culture was added, followed by either 100 *μ*L or 10 *μ*L wash solution. Soft TSB agar tubes were then poured on TSA plates supplemented with nalidixic acid. Formation of plaques after incubation at 35 ± 0.5°C for 18–24 h was counted to obtain CFU·g^−1^ counts.

Additional controls were prepared using both a high titer of Phi X-174 (DSM-4497) and a negative control (without any phage) to confirm specificity of* E. coli* CN-13 and to ensure no phage contamination occurred when propagating the host culture. Controls were incubated in conditions similar to the wash samples.

### 2.3. Produce Analysis: Molecular Method

Isolates were obtained from the confirmed positive samples in the culture method (atypical colonies on MCA plates for* E. coli* or XLD agar plates for* Salmonella* spp.). For DNA extraction of these isolates, the culture stocks underwent boil lysis method following the protocol of de Medici et al. [12] with modifications. Briefly, 0.05 N NaOH was added and boiled at 95°C for 15 min. The DNA extracts were then kept at −20°C and transferred to International Environmental Analysis and Education Center, Gwangju Institute of Science and Technology, Korea, in an ice box for molecular analysis.

Confirmation of bacterial isolates was done using TaqMan real-time PCR (RT-PCR) with amplification of DNA performed using a Rotor-Gene 3000 (Corbett Research) real-time PCR instrument. TaqMan Environmental Master Mix (Applied Biosystems, USA) was used for the RT-PCR assay of* E. coli*, with primers specific for* E. coli* adapted from Takahashi et al. [13]. The forward primer ECN1254F (5′-GCA AGG TGC ACG GGAATA TT-3′) and reverse primer ECN1328R (5′-CAG GTG ATC GGA CGC GT-3′) employed in this study amplify the* uidA* gene (*β*-glucuronidase). The dual labelled probe, ECL1277p (5′-CGCCACTGGCGGAAGCAACG-3′), was added to the reaction.

Real-time PCR assay of* Salmonella *spp. was conducted using AccuPower Plus DualStar qPCR Premix (Bioneer, Korea), and primers for this assay were adapted from Elizaquível and Aznar [14]. The forward primer OriP1 (5′-TTA TTA GGA TCG CGC CAG GC-3′) and reverse primer OriP3 (5′-GGA CCA CGA TCA CCG ATC A-3′) amplify the* OriC* gene (replication origin sequence). The dual labelled probe, OriP214 (5′-TCA ATG CGT TGG AAA GGA TCA CTA GCT GT-3′) was included to the reaction.

*E. coli* ATCC strain 15597 and* Salmonella* KCTC strain 2421 were used as standards in this part of the experiment. They were grown in TSB as a simple enrichment medium, serially diluted in 0.1% BPW, and spread plated on TSA plates to enumerate the sample as CFU/mL. DNA was extracted from these standards and run in RT-PCR instrument.

The 20 *μ*L reaction consisted of 2x RT-PCR Mastermix, 0.5 *μ*M of forward and reverse primers, 0.25 *μ*M of probe, 0.1 *μ*g/*μ*L bovine serum albumin, and 2 *μ*L of DNA template. Each set of samples assayed included a nontemplate control (NTC) and DNA standards in duplicate. RT-PCR for* E. coli* was performed with an initial denaturation at 95°C for 15 min, followed by 40 cycles at 95°C for 15 sec, and 63°C extension step for 60 sec where the fluorescent signal was acquired. For reactions with* Salmonella* isolates, the conditions had an initial denaturation step at 95°C for 5 min, followed by 40 cycles at 95°C for 15 sec, and 63°C extension step for 60 sec. All samples with fluorescent signals at the end of 40 cycles compared with no signal from the negative controls were scored as positive.

### 2.4. Statistical Analysis

Data gathered from the microbiological assessment of fresh produce were subjected to single-factor analyses of variance (ANOVA) using the General Linear Model Procedure (PROC GLM) of the SAS statistical package version 9.1.3 [15] with Duncan Multiple Range Test (DMRT) for post hoc determinations of significant differences between the five open air markets and between open air markets and supermarkets (*α* = 0.05).

## 3. Results

A total of 300 fresh produce samples eaten raw, namely, bell pepper, cabbage, carrot, lettuce, and tomato from five open air markets and five supermarkets, were surveyed for their microbial quality in the Philippines using culture technique and confirmed by molecular analysis.* E. coli* isolates were observed in 50 of the 300 fresh produce samples surveyed. Further, of the 300, 74 produce samples were found contaminated with* Salmonella* spp., and 141 samples recovered somatic phage from produce wash solutions.  Table 1 shows the typical ranges of results of microbial contamination that were observed in fresh produce.

Thermotolerant* E. coli* had values as high as 4.15 log_10_ CFU/g, while* Salmonella* spp. values were observed as high as ≥2.34 log_10_ MPN/g determined when all MPN dilution wells were positive after incubation in RV. All positive isolates were confirmed through RT-PCR using fluorescent probes specific for these microorganisms. For somatic phage, most values are found to be higher than 3.00 log_10_ PFU/g, with plaques being too numerous to count even with 10 *μ*L sample wash solution being used for the assay (Table 1).

Table 2 shows the prevalence of* E. coli*,* Salmonella,* and somatic phages in the fresh produce surveyed. All samples positive for* E. coli *(50 isolates) and* Salmonella *(74 isolates) spp. were subjected to RT-PCR for confirmation. RT-PCR revealed fluorescent signals at the end of each cycle for positive isolates. It was observed that carrots had the highest microbial counts. This may be attributed to environmental factors such as the manner of planting and harvesting. Soil can greatly contaminate produce, transferring the microorganisms from environment to produce [16]. Pre- and postharvest processes utilizing contaminated water may also contribute to contamination. On the other hand, tomatoes had the least number of microbial contaminants in both open air and supermarkets. This could be due to its smooth pericarp that may possibly inhibit attachment of microorganisms.

Open air markets were observed to have statistically similar* Salmonella* spp. and somatic phages counts, except for* E. coli* (Table 3). Post hoc analysis of open air markets showing* E. coli* contamination revealed that these markets formed two groups, with markets from the provinces in South and Central Luzon forming one cluster and markets from the National Capital Region forming another group.

Although supermarkets are thought to have more stringent processing prior to vending, all log values were statistically insignificant between open air and supermarkets (*P* ≤ 0.05) (Table 3). The data suggest that incidence of microbial contamination among markets and supermarkets are likely similar.

Varying molecular techniques can be utilized for the detection of coliforms. While immunological techniques have shown promise for their detection, there are some problems of low antibody specificity. PCR has shown more promise in the detection of coliform bacteria in both sensitivity and specificity [17–19]. The confirmation by RT-PCR revealed fluorescent signals for all positive isolates compared to a lack of signal in the nontemplate controls, while positive controls also indicated expected amplification (data not shown). Additionally, the specificity of the RT-PCR primers and probes confirms the results of the culture techniques that were employed in this study.

## 4. Discussion

Consumption of fresh produce from health conscious consumers has resulted in a growing industry with global production for fruits and vegetables increasing from 1979 to 2004 [20]. However, it is facing new challenges that require attention, such as the protection of consumers against food-borne pathogens [21]. Contamination of vegetables may occur during growth, harvest, or processing. Under certain conditions, microorganisms can also become localized within vegetables. Conditions that promote entry of microorganisms include damage to the natural structure such as punctures, stem scars, cuts, and splits [22]. In the past several decades, there has been an increase in the occurrence of food-borne illness linked to fresh fruits and vegetables. Fresh vegetables and herbs including those of the leafy variety have been attributed as vehicles for the transmission of microbial food-borne disease worldwide [23]. For instance, in the USA between 1998 and 2002, vegetables were associated with 2.9% (192/6647) of the total food-borne outbreaks recorded [24].

The target bacteria and phage studied in this survey were bacterial pathogens, potentially opportunistic pathogens, or indicator organisms for fecal contamination.* E. coli *is a Gram-negative, rod-shaped bacterium commonly found in the gastrointestinal tract and is also one of the most implicated pathogens on diarrheal cases worldwide [25, 26]. Leafy vegetables are commonly linked to food-borne infections where* E. coli* serves as the responsible disease agent [27–29]. As shown in this study, a number of lettuces and cabbages were contaminated with* E. coli*. Gilbert et al. [30] reviewed the dose-response estimates for* E. coli* O157:H7 and original estimates of infectious dose were less than a few hundred cells. While* E. coli* isolates obtained in this study have not been identified as pathogenic strains, they demonstrated the ability to be cultured at high temperature of 44.5°C for 24 h in relatively high prevalence (Tables 1 and 2).

*Salmonella* spp. are the most commonly identified aetiological agent associated with fresh produce-related infection, isolated in 48% of cases between 1973 and 1997 in the USA [31] and in 41% of cases during 1992–2000 in the UK. A range of fresh fruit and vegetable products have been implicated in* Salmonella* infection, most commonly lettuce, sprouted seeds, melon, and tomatoes [32]. Jay et al. [33] included data on the incidence of salmonellae in fruit, vegetables, and spices with the prevalence shown to be below 10%. They noted that numbers of salmonellae on raw vegetables are usually <1 log_10_ CFU/g. On the other hand, this study shows that* Salmonella* can be as high as 2.34 log_10_ MPN/g (Table 2). The high detection rate of* Salmonella* can be alarming as many food-borne outbreaks have been associated with this microorganism (Table 2).

Additionally, studies for the impact of enteric viruses on produce in Southeast Asian countries are also limited. While direct detection of enteric viruses would be problematic due to the intensive processing needed (molecular methods and cell culture), a possible alternative was utilized in this study, surveying the presence of somatic bacteriophages [34]. Such bacteriophages have served as ideal indicators of enteric viruses for water contamination [35] and could serve as model organisms for virus contamination of agricultural produce. Enteric viruses have a low infective dose and remain active even after exposure to low pH (<3) and temperature extremes [36]. While somatic phages are not pathogens, they are classic indicator organisms for fecal contamination and can be utilized to mimic and, in turn, serve as a predictor for the survival of enteric viral pathogens on produce. This study is the first in the Philippines to present and investigate somatic bacteriophages in a wide array of fresh produce.

Gabriel et al. [37] surveyed the microbiological composition of one produce sample, mung bean sprouts, vended in public markets from the National Capital Region; however, only* Salmonella* spp. were observed in that study. Additionally, the data presented were only qualitative in nature and reported as the number positive (or negative) per 25 g samples [37]. In contrast, this study has shown that there is high prevalence of bacterial pathogens and fecal indicator organisms from various fresh produce samples from both open air and supermarkets (Tables 1 and 2). Furthermore, these microbes are not only observed for their presence but also quantified and compared.

This study observed that carrots had the highest microbial counts and may be attributed to environmental factors from soil as there is extensive exposure during cultivation of this produce type. Soil can greatly contaminate produce, transferring the microorganisms from environment to produce [16]. Pre- and postharvest processes utilizing contaminated water may also contribute to contamination. Alternately, tomatoes had the least number of microbial contaminants in both open air and supermarkets which could be due to its smooth pericarp that may possibly inhibit attachment of microorganisms.

It is also interesting to note and compare the prevalence of bacterial pathogens and phages in the two types of markets. Although produce samples from supermarkets are thought to be less contaminated due to their processing, all log values were found to be statistically similar between open air and supermarkets (*P* ≤ 0.05) (Table 3). The data suggest that microbial contamination from all types of markets and supermarkets has a similar prevalence despite the differences in operation.

It is important to note that confirmation of bacterial isolates was done using TaqMan RT-PCR which is highly specific. This method has several advantages as identification of bacterial isolates can be rapid and accurate. It has been demonstrated that RT-PCR assay can not only be specific, but also have amplification kinetics suitable for the specific detection of* E. coli*, irrespective of strain [13]. This study investigated the presence of the* uidA* gene, which is encoded for *β*-D glucuronidase and is found in almost all strains including the pathogenic strains,* E. coli* O157:H7 [14], and also demonstrated the specificity of OriP1/P3 primers and OriP214 probe for* Salmonella* spp. [14]. While enumeration of such bacterial organisms in this study used simple culture techniques such as multiple-tube fermentation and membrane filtration, these routine methods have limitations. Potential growth of heterotrophic microbial flora from the samples despite using selective media and lack of specificity are some issues. Hence, the use of PCR incorporating a probe was ideal for rapid, sensitive, and specific confirmation of bacterial isolates.

The usefulness of the microbial indicators as tools for risk assessment can be significantly enhanced by the development of testing methods and analysis techniques that can define specific sources of these organisms. In particular, real-time PCR has several advantages including enhanced speed and the absence of post-PCR processing steps. Rapid and accurate identification of bacterial pathogens from food samples is important, both for food quality assurance and for tracing outbreaks of bacterial pathogens within the food supply. Growing concerns regarding the safety of fresh produce warrant a greater emphasis on the development of more rapid, specific, and highly sensitive detection methods [38]. It was shown in this study that RT-PCR can be used as a rapid method in confirming bacterial isolates and shows potential for using similar techniques for determining microbial contamination in fresh produce.

In the Philippines, policies that can help reduce microbial contamination of foods are strengthened by the Department of Agriculture (DA) due to the country's high dependence on agriculture [39, 40]. The Good Agricultural Practice for Fruits and Vegetable Farming (GAPVF) is a set of consolidated safety and quality standards formulated by the DA for on-farm fruit and vegetable production. These codes of practices are based on concept of Hazard Analysis of Critical Control Points (HACCP) and quality management principles. The basis of GAPVF program is to provide safe food product for the consumers and its focus is to reduce risk of microbial and pesticide contamination. Additional benefits of the program are worker safety and protection of the environment. However, the current technologies employed cannot absolutely eliminate food safety hazards associated with fresh produce which are to be consumed raw, as shown in the data and results from this study.

## 5. Conclusions

This study is the first comprehensive survey of microbial contamination of fresh produce in the Philippines. The increasing awareness of Filipinos of a healthy diet, that is, consuming fresh produce, may also pose an unintentional risk of increasing the incidence of gastrointestinal illnesses (and other related diseases) by the consumption of contaminated foods. The recent undocumented food outbreaks in the country also make it timely to investigate the prevalence of bacterial pathogens and phages in food. Thus, it is imperative to conduct surveillance on these produce samples for microbial contamination to educate consumers buying either in open air markets or in supermarkets resulting in disease detection and prevention. Further, the results established in the study may be of use to farmers, retailers, food safety educators, and policy makers in improving the microbiological quality and safety of fresh produce in the Philippines and in preventing the occurrence of diseases associated with it.

## Figures and Tables

**Table 1:** Microbiological quality of fresh produce from open air and supermarkets in the Philippines^a^.

Markets and fresh produce	*E. coli* (log_10_⁡CFU/g)	*Salmonella* spp. (log_10_⁡MPN/g)	Coliphages (log_10_⁡PFU/g)
Open air markets			
Bell pepper	1.62–3.95	0.66–2.34≤	2.15–3.00≤
Cabbage	0.30–2.58	0.66–2.34≤	1.41–3.00≤
Carrot	2.30–4.03	0.66–2.34≤	2.41–3.00≤
Lettuce	1.20–3.92	0.66–2.34≤	1.60–3.00≤
Tomato	0.88–3.66	0.66–2.34≤	3.00
Supermarkets			
Bell pepper	2.26–4.15	0.66–2.34≤	1.30–3.00≤
Cabbage	1.00–2.88	0.66–1.68≤	1.30–2.15
Carrot	1.75–2.78	0.66–2.34≤	1.60–2.15
Lettuce	3.09–3.15	0.66–2.34≤	1.30–3.00≤
Tomato	1.94–3.12	0.00	0.00

^a^Values are ranges of 50 fresh produce samples per type from all open air markets; 10 fresh produce samples per type from all supermarkets; a total of 300 fresh produce samples.

**Table 2:** Prevalence of microbial contamination in fresh produce from open air markets and supermarkets in the Philippines by culture and real-time PCR techniques^a^.

Fresh produce	*E. coli *		*Salmonella* spp. (% and no. of positive/total)		Coliphages	
	Open air	Supermarket	Open Air	Supermarket	Open air	Supermarket
Bell pepper	10% (5/50)	20% (2/10)	20% (10/50)	30% (3/10)	62% (31/50)	60% (6/10)
Cabbage	10% (5/50)	20% (2/10)	22% (11/50)	10% (1/10)	52% (26/50)	60% (6/10)
Carrot	22% (11/50)	30% (3/10)	38% (19/50)	50% (5/10)	38% (19/50)	100% (10/10)
Lettuce	24% (12/50)	20% (2/10)	24% (12/50)	30% (3/10)	64% (32/50)	80% (8/10)
Tomato	16% (8/50)	0% (0/10)	18% (9/50)	10% (1/10)	6% (3/50)	0% (0/10)

Total	41/250	9/50	61/250	13/50	111/250	30/50

^a^Values are expressed in percentages of contaminated fresh produce from 60 per type from all open air markets, 10 fresh produce samples per type from all supermarkets. Values in parentheses are number of positive samples for *E. coli*, *Salmonella* spp., and somatic phages over the total produce surveyed.

**Table 3:** Statistical analysis of microbial quality of fresh produce collected from open air and supermarkets in the Philippines.

Microorganisms	Difference between open air markets	Difference between open air and supermarkets
*E. coli *	0.0001^a^	0.9147
*Salmonella* spp.	0.1280	0.3229
Somatic phage	0.2152	0.8161

^a^Value is statistically significant at *α* = 0.05.
